# Electrodeposition of Aluminum Coatings from AlCl_3_-NaCl-KCl Molten Salts with TMACl and NaI Additives

**DOI:** 10.3390/ma13235506

**Published:** 2020-12-02

**Authors:** Tianyu Yao, Haiyan Yang, Kui Wang, Weiping Wu, Haiyan Jiang, Hezhou Liu, Qudong Wang, Wenjiang Ding

**Affiliations:** 1 National Engineering Research Center of Light Alloy Net Forming, Shanghai Jiao Tong University, Shanghai 200240, China; yty123261002@sjtu.edu.cn (T.Y.); jianghy@sjtu.edu.cn (H.J.); wangqudong@sjtu.edu.cn (Q.W.); wjding@sjtu.edu.cn (W.D.); 2 The State Key Lab of Metal Matrix Composites, Shanghai Jiao Tong University, Shanghai 200240, China; hzhliu@sjtu.edu.cn; 3 Department of Electrical and Electronic Engineering, School of Mathematics, Computer Science and Engineering, City, University of London, Northampton Square, London EC1V 0HB, UK; weiping.wu@city.ac.uk

**Keywords:** TMACl, NaI, electrodeposition, molten salts, Al coating, additive

## Abstract

The Al coatings achieved via electrodeposition on a Cu electrode from AlCl_3_-NaCl-KCl (80–10–10 wt.%) molten salts electrolyte with Tetramethylammonium Chloride (TMACl) and Sodium Iodide (NaI) additives is reported. The effect of the two additives on electrodeposition were investigated by cyclic voltammetry (CV), chronopotentiometry (CP), scanning electron microscopy (SEM) and X-ray diffraction (XRD). Results reveal that compact and smooth Al coatings are obtained at 150 °C by the electrodeposition process from the electrolyte with 1% TMACl and 10% NaI. The Al coatings exhibit great corrosion resistance close to that of pure Al plate, with a corrosion current of 3.625 μA. The average particle size is approximately 2 ± 1 μm and the average thickness of the Al layer is approximately 7 ± 2 μm. The nucleation/growth process exhibits irrelevance with TMACl or NaI during the electrodeposition of Al. TMACl cannot affect and improve the electrodeposition effectively. However, the addition of TMACl and NaI can intensify the cathodic polarization, producing an inhibition of Al deposition, and contribute to form uniform Al deposits. This can increase the conductivity and facilitate in refining the size of Al particles, contributing to forming a continuous, dense and uniform layer of Al coating, which can be used as effective additives in molten salts electrolyte.

## 1. Introduction

High temperature electrolysis in molten salts is an important industrial process across many important areas. In industry, it has been widely used for metals extraction, materials processing and metallic thin film deposition. Over the years, the Hall–Héroult process has been used for the smelting of pure aluminum (Al) at industrial scale [1], the most widely used non-ferrous metal in the world. Al oxide (alumina, Al_2_O_3_) dissolved in molten cryolite (Na_3_AlF_6_) and consumable carbon are used as anodes in the process. The electrolysis in molten salts is not energy efficient, consuming huge amount of energy and carbon materials (11.5–13.5 kWh electricity and 0.4–0.5 kg carbon anodes for producing 1 kg Al [2]). Al production is highly energy-intensive, more than 3% of the world’s entire electrical supply has been used to extraction of Al every year [3]. The primary Al industry induces 0.4 billion tons (Gt) of carbon dioxide equivalent (CO2e) greenhouse gas emissions every year [4]. As the inorganic salts (fluorides, chlorides and carbonates melts) have high melting points, the process requires high temperature conditions and consumes large amount of thermal energy [5]. It is essential and important to develop a low temperature, energy efficient and low carbon footprint for sustainable electrolysis processes in molten salts, for economical, safety and environmental considerations.

Electrodeposition of Al coatings has drawn much attention because of their excellent properties, i.e., low density, resistance to corrosion and high-temperature oxidation. Compared with other methods of preparing Al coatings [6,7,8,9,10], electrodeposition shows a lot of advantages: (1) the convenient and easy operation, (2) higher cost performance, (3) suitable for substrates of various geometric shapes, (4) quality and thickness of the coatings could be tuned through adjusting the processing parameters of electrodeposition.

Among all categories of nonaqueous electrolyte systems used for Al electrodeposition, AlCl_3_-NaCl-KCl molten salts is becoming a popular candidate for electroplating Al coatings and has been frequently adopted in the electro-deposition of Al and Al alloys on account of their low eutectic point [11]. A lot of researches have been developed on the electro-deposition of Al in AlCl_3_-NaCl-KCl molten salts [12,13,14,15], some binary or ternary alloys were successfully synthesized by incorporating different metal ions [16,17,18,19,20] into the electrolytes. Different substrate materials, i.e., graphite [21], Au [19], were also investigated. However, the poor quality of Al coatings electrodeposited from AlCl_3_-NaCl-KCl molten salts is still a pressing issue. The dendrites of Al and microholes forming during the electrodeposition constrains the uniformity of Al coatings severely. Particularly, it is the dendrites and microholes that make the Al coatings more vulnerable to rupture and detach off the substrate with the increasing thickness of Al deposition. This challenge severely impedes the further applications of Al or Al alloy coatings prepared by electro-deposition.

In order to prepare high-quality Al coatings, appropriate additives in the electrolytes are essential [22,23]. Additives are widely used in metal electrodeposition in aqueous solutions, while, only a few studies pay attention to additives in the electrolytes for Al electrodeposition in nonaqueous solutions, especially in inorganic molten salts [24,25]. Generally, different types of additives serve different electrolytes, and the solubility in the electrolyte has to be taken in consideration. Amines [26], pyridines, nicotinamides [27] and other small molecule compounds are the proper candidate additives in organic solvents and ionic liquids electrolytes. In contrast, the additives added in molten salts electrolytes could be some small ionic and inorganic molecules. Previous researches on additives showed that some halides [12], especially alkali halides [24,25] could be adopted to improve the quality of coherent and dendrite-free Al coatings. Hydrogen halides are too difficult to handle, since they are usually liquid and volatile. Alkali halides could be ideal alternatives when used as remarkable additives since they exhibit good solubility and low volatility. LiCl and KCl had been proven to be helpful to improve the quality of Al coatings [22,23]. KI had also been found an effective surfactant in electrodeposition of Al from AlCl_3_-NaCl-KCl molten salts [28,29]. The addition of NaI showed improvement in the quality of deposited Al layers [30]. The authors’ researches also demonstrated that NaI could intensify cathodic polarization, inhibit growth of Al deposits and facilitate the formation of uniform Al deposits [31].

The selected additives have improved the quality of electrodeposition Al coatings remarkably. While, the destruction of the AlCl_3_-NaCl-KCl molten salts electrolyte due to the volatility of AlCl_3_ is still another severe problem, which restricts the efficiency of the electrodeposition. The temperature of Al electrodeposition in AlCl_3_-NaCl-KCl molten salts should be set at the range of 130~250 °C. While, the AlCl_3_ in the molten salt’s electrolyte volatile seriously, so that the composition of AlCl_3_-NaCl-KCl changes gradually, leading to an inevitable degradation of Al coating quality. In order to promoting the quality of the electrodeposition coatings, some additives are also adopted to improve the electrolyte [32]. According to the reactions in AlCl_3_-NaCl-KCl molten salts [31], as some macromolecular cations are introduced into the electrolyte, facilitating the equilibrium shift. It is expected to reduce the volatility of the electrolyte, improve electrolyte stability and further improve the coating quality.

As one important type of halides, ammonium halides have seldom been studied as additives in molten salts electrolytes. In industry, ammonium salts are widely used as effective cationic surfactants. It is supposed that the combination of two different types of additives, alkali halides and ammonium halides, could contribute to improving the coating quality and prolonging the electrolyte service life. In fact, in order to understand the mechanism comprehensively and achieve better Al coatings, electrochemical process and nucleation/growth mechanisms are of a great benefit. Since single additive is effective, it is reasonable to predict that the addition of multiple additives could improve the quality of Al coatings, as the sum is always greater than the parts. However, the effects of multiple additives have not been investigated yet.

In the present study, we have introduced the ammonium halides as the additives in molten salts electrolytes for the low temperature (150 °C) electrodeposition of Al. We are focusing on two specific halides—TMACl (Tetramethylammonium Chloride) was adopted to reduce the volatility of the electrolyte and NaI (Sodium Iodide) was adopted to improve the coating quality. The effects of TMACl and NaI on the deposition mechanisms, the morphology and microstructures of Al from AlCl_3_-NaCl-KCl (80–10–10 wt.%) molten salt electrolyte were studied systematically. Cyclic voltammetry (CV) and chronoamperometry (CP) have been employed to investigate the electrochemical process and the deposition mechanisms of Al. Electrodeposits were characterized using scanning electron microscopy (SEM) and X-ray diffraction (XRD). The corrosion resistance of Al coatings was tested through potentiodynamic polarization (PD) and electrochemical impedance spectroscopy (EIS).

## 2. Materials and Methods

### 2.1. Electrolytes Preparation

Sodium chloride (NaCl, AR, 99.9% purity, Sinopharm, Beijing, China) and potassium chloride (KCl, AR, 99.9% purity, Sinopharm) were initially dried in a vacuum oven for 72 h at 300 °C. Anhydrous Al chloride (AlCl_3_, AR, 99.8% pure, Sinopharm), NaI (AR, 99.5%, Macklin, Shanghai, China) and TMACl (AR, 99%, Macklin) were used as received. AlCl_3_, NaCl and KCl (80–10–10 wt.%) were mixed and melted at 150 °C in an electrolytic cell on a heating device with temperature control system. The mixture was purged with high-purity argon (Ar) gas. All of the electrodeposition and electrochemical experiments were performed in the glove box (Mikrouna Upure 1220/750/900, Shanghai, China). In order to investigate the effect of NaI and TMACl on the electrodeposition of Al, NaI, TMACl or their mixture were added into the electrolytes and the electrolyte continuously stirred for 4 h to yield a homogeneous yellowish liquid.

### 2.2. Electrolyte Volatility Tests

The electrolyte volatility tests were performed after the electrolyte had been well prepared in the glove box. The temperature-controlled heater maintained the electrolytic system at 150 °C. When we opened the lid of the electrolytic cell, the AlCl_3_ started to spill out of the electrolytic cell. Then, we weighed the mass of the electrolytic cell system every 30 min, where the mass loss over the fixed period of time could be calculated as the volatile mass of AlCl_3._ The electrolyte volatility tests lasted for 4 h.

### 2.3. Electrochemical Tests

An electrochemical workstation (CHI655D, Chenhua, Shanghai, China) was adopted in all electrochemical experiments. A typical three-electrode cell was used in the experiments, a glassy carbon (GC, 0.07 cm^2^) was adopted using as a working electrode and an Al plate (99.99%, 350 mm^2^) was used as a counter electrode. A pure Al bar (99.99%, diameter Φ = 0.5 mm) was adopted as the reference electrode, and it was fixed in a small glassy tube which was full of the AlCl_3_-NaCl-KCl (weight ratio 8:1:1) molten salts. The Al plate and the pure Al bar were burnished with abrasive paper, cleaned in an ultrasonic bath for 3 min, rinsed with distilled water, then dried in a vacuum oven before all the experiments and characterizations. Cyclic voltammogram (CV) tests were carried out with a scan rate of 50 mV/s.

### 2.4. Electrodeposition

In the electrodeposition process, the Al bar described above was adopted as the reference electrode. The Al plate described in the last section was used as anode. A pure Cu foil (the area exposed to the solution is 5 mm × 5 mm, 99.9%) was adopted as the cathode. The Cu foil, Al plate and Al bar were all cleaned with distilled water and then dried in a vacuum oven. The electrodeposition of Al was performed at 150 °C under a constant current mode. Current density was kept at 50 mA/cm^2^, and the electrodeposition process lasted for 10 min. The samples were cleaned after the electrodeposition using an ultrasound bath for 3 min, rinsed with distilled water and then dried in a vacuum oven.

### 2.5. Characterization of the Al Coatings

A field emission scanning electron microscope (FE-SEM, SIRION200, FEI, Shanghai, China) was used to observe the surface and cross-sectional morphologies of the Al coatings. The samples were examined by X-ray diffraction (XRD, D/MAX2000V, Rigaku, Tokyo, Japan) to identify the constituents and the crystal structure by the 2θ/θ scanning mode. The data was collected in a 2θ = 30°−90° range with a scanning speed of 5° min^−1^ with 0.01° (2θ) step size.

Electrochemical behavior was studied in the 3.5 wt.% NaCl solution at room temperature by potentiodynamic polarization to evaluate the corrosion resistance. A graphite electrode and a saturated calomel electrode (SCE) were used as the counter electrode and the reference electrode, respectively. All the specimens were held at open circuit for 30 min to reach the steady value prior to potentiodynamic polarization. Scans were obtained from 100 mV below open circuit potential (OCP) to and scanned upwards at a rate of 0.5 mV/s. A PARSTAT 2273 advanced electrochemical system from Princeton Applied Research (New York City, State of New York, USA) was adopted to perform the electrochemical impedance spectroscopy (EIS) tests. The EIS tests conducted at OCP with a sinusoidal signal amplitude of 10 mV. The frequency ranged from 10^5^ Hz down to 1 Hz.

## 3. Results and Discussion

### 3.1. Electrolyte Volatility

**Figure 1:**
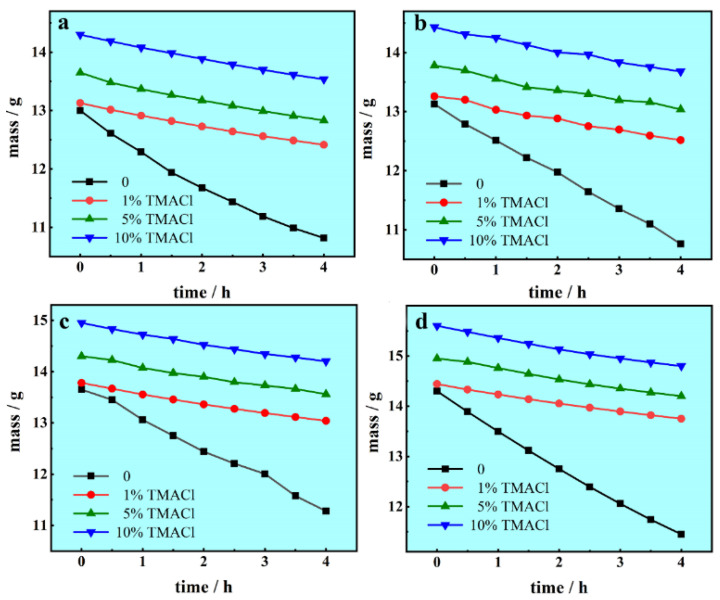
The mass-time transients of the electrolyte volatility experiments in the AlCl_3_-NaCl-KCl molten salts electrolyte, (**a**) no additive, (**b**) 1 wt.% NaI, (**c**) 5 wt.% NaI, (**d**) 10 wt.% NaI, at 150 °C.

To study the effect of TMACl on the volatility of AlCl_3_-NaCl-KCl molten salts electrolyte, the volatilization experiments were performed and the results are shown in Figure 1. It can be seen that the AlCl_3_-NaCl-KCl molten salts electrolyte volatilized continuously at 150 °C. Figure 1a shows the mass of volatile AlCl_3_ could be as much as 2.180 g after a 4 h exposure. However, when TMACl was added into the electrolyte, the volatility of the electrolyte declined dramatically. As 1 wt.% TMACl, 5 wt.% TMACl, 10 wt.% TMACl was brought into the electrolyte, the mass of volatile AlCl_3_ was 0.716, 0.818 and 0.765 g, respectively. The addition of TMACl could effectively inhibit the volatilization of AlCl_3_, and more TMACl in the electrolyte could not further reduce the volatility.

Different contents of NaI were added to test whether TMACl could inhibit the volatilization of AlCl_3_ in the electrolyte with NaI. As shown in Figure 1b–d, the mass of volatile AlCl_3_ effectively declined after the addition of TMACl, proving that the effect of inhibiting the volatilization of TMACl could not be impaired by NaI. Different content of TMACl had the similar effect on the volatilization of AlCl_3_. A reasonable explanation of the effect of TMACl is that the TMA^+^ ionized from TMACl formed complex ions with Al_2_Cl_7_^−^, preventing Al_2_Cl_7_^−^ from forming AlCl_3_.

### 3.2. Voltammetric Behavior

The cyclic voltammograms (CV) of Al electrodeposition on the glassy carbon electrode in the AlCl_3_-NaCl-KCl (80–10–10 wt.%) molten salts electrolyte at 150 °C has been studied, as shown in Equation (1), only one electrodeposition reaction occurred according to the composition of the electrolyte in which the main type of ions is Al_2_Cl_7_^−^:

(1) $$4{\mathrm{Al}}_{2}{{\mathrm{Cl}}_{7}}^{-}\;+\;3e^{-}\;⇆\;\mathrm{Al}↓\;+\;7{{\mathrm{AlCl}}_{4}}^{-}$$

The crossing current loop and the oxidation/reduction peaks demonstrate that the electrodeposition of Al in AlCl_3_-NaCl-KCl molten salts is irreversible.

**Figure 2:**
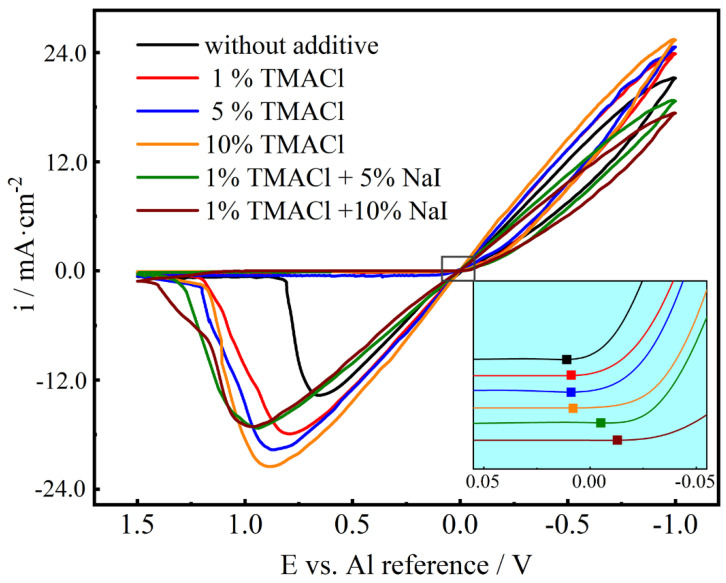
The cyclic voltammograms of the AlCl_3_-NaCl-KCl molten salts electrolyte without additive, 1% TMACl, 5% TMACl, 10% TMACl, 1% TMACl + 5% NaI and 1% TMACl + 10% NaI, at 150 °C.

The CV test was performed in AlCl_3_-NaCl-KCl (80–10–10 wt.%) molten salts electrolyte with various amount of the two additives on the glassy carbon electrode in order to study the effect of NaI and TMACl on the voltammetric behavior of Al. The CV curves in AlCl_3_-NaCl-KCl molten salts electrolyte without additive, with 1% TMACl, 5% TMACl, 10% TMACl, 1% TMACl + 5% NaI and 1% TMACl + 10% NaI are presented in Figure 2.

Every cyclic voltammogram was recorded on a fresh glassy carbon electrode surface. It shows that the cathode deposition current density increased with the addition of TMACl, indicating that TMACl cannot give rise to an inhibition of Al deposition. However, as both NaI and TMACl were added into the electrolyte, the deposition current density decreased dramatically. The related experiments [31] proved that the addition of NaI decreased the cathodic current density and gave rise to an inhibition of Al deposition. A possible reason is that the introduction of two additives leads to plenty conductive ions in the molten salts, and the TMACl does not impair the effect of NaI. As shown in the inset of Figure 2, the reduction potential of cathodic was stable at approximately 0.01 V with or without the increase of TMACl, which indicates the addition of TMACl has no prominent effect on changing the reduction potential of cathodic. While, the reduction potential of cathodic shifted obviously in a negative direction after the NaI was added into the electrolyte. The results showed that the addition of both TMACl and NaI intensified the cathodic polarization, while the addition of TMACl could not, implying the polarization effect might be caused by NaI. Generally, appropriate polarization is necessary for electrodeposition as high overpotential is needed for the nucleation. The addition of TMACl and NaI intensifies the polarization and contributes significantly to the formation of better Al coatings.

Additionally, the addition of the additives made the anodic current density peak shift to the positive direction in different extent, the potential difference (∆*E*_P_) [33] between oxidation and reduction became increasingly large. This means that the two additives could aggravate the irreversibility in the electrodeposition system [34]. The physical properties of the electrolyte do not change with the addition of two additives, the SEM, EDXS and XRD characterizations proved that the two additives could not be introduced in Al deposit as the impurities.

### 3.3. Chronoamperometric Investigations

**Figure 3:**
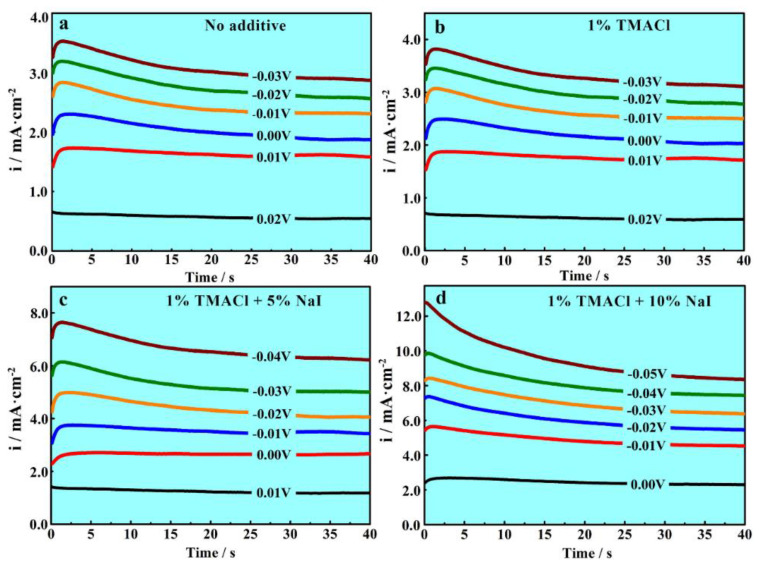
Typical current density–time transients of the electrodeposition experiments in the AlCl_3_-NaCl-KCl molten salts electrolyte, (**a**) no additive, (**b**) 1% TMACl, (**c**) 1% TMACl + 5% NaI and (**d**) 1% TMACl + 10% NaI, at 150 °C.

Chronoamperometric experiments were conducted on a glassy carbon electrode on purpose of further studying the effect of TMACl and NaI on Al nucleation and growth mechanism in AlCl_3_-NaCl-KCl molten salts electrolyte. The experiments started soon after the short induction times, and the potential started at the value where no reduction of Al(III) took place, and then the potential increased gradually, to a more negative value which was much more negative than the nucleation and growth of Al particles occurred. The experiments were performed in the AlCl_3_-NaCl-KCl molten salts electrolyte with TMACl and NaI at different content densities, and four typical representative current-time transients are plotted in Figure 3.

Irrespective of the different content of additives, the shapes of the peaks in the potential step current density transients are closely related to nucleation/growth process. An obvious augment owing to the formation and growth of aluminum nuclei in each current curve can be easily observed. Additionally, the process of formation and growth made Al ions collect rapidly. As the overlap [35] activates, the increasing current density receives the maximum, i_m_, and the time the peak appears is the time, t_m_. After that, all of the current density transients tend to decrease because of the consumption and diffusion of Al_2_Cl_7_^−^. With the applied potential increases, the i_m_ becomes higher, while the t_m_ becomes shorter at the same time. It indicates that the time of overlapping decreased as the nucleation density rises. In addition, the current density transients are not going to converge to the same value, the stable values of current density increase as the potential applied increases. This proves that the electrodeposition of Al in AlCl_3_-NaCl-KCl molten salts electrolyte is the electrochemical reaction controlled. The reactions determine the main ions in the AlCl_3_-NaCl-KCl molten salts electrolyte are Al_2_Cl_7_^−^ ions, and abundant Al_2_Cl_7_^−^ ions count for the electrochemical reaction control. Diffusion control is usually observed in most ionic liquids due to the lack of reductive ions near the cathode electrode [36].

As the different content of additives were taken into consideration, it was found that the addition of TMACl and NaI made the potential at which the reduction of Al^3+^ started shift to negative direction. On the contrary, the addition of TMACl did not work effectively. The result illustrated that the NaI could increase the overpotential of electrodeposition and promotes the cathodic polarization, but TMACl could not, which agreed well with those of the above voltammograms. I^−^ ions ionized from NaI might act as some surfactant and the adsorption of I^−^ on the surface of the electrode benefits nucleation and inhibits reduction of Al^3+^ [34]. It also revealed that additives produced an increase in response current and this effect increases with the concentration of the additive, whether TMACl or NaI. It is probably because the addition of the two additives increases the number of charged particles and activity of the electrolyte, so that the electrical conductivity increases and the response current rises. I^−^ ions would impede active sites on the surface of cathode, increasing the number of growing nuclei. The I^−^ ions inhibit the growth of nuclei, which lead to an energetic homogenization of the Al coatings. While, the Cl^−^ ions and ammonium ions ionized from TMACl could not, which implies TMACl is not a suitable additive in the AlCl_3_-NaCl-KCl molten salts electrolyte. The combined addition of TMACl or NaI would help improve the Al coatings.

**Figure 4:**
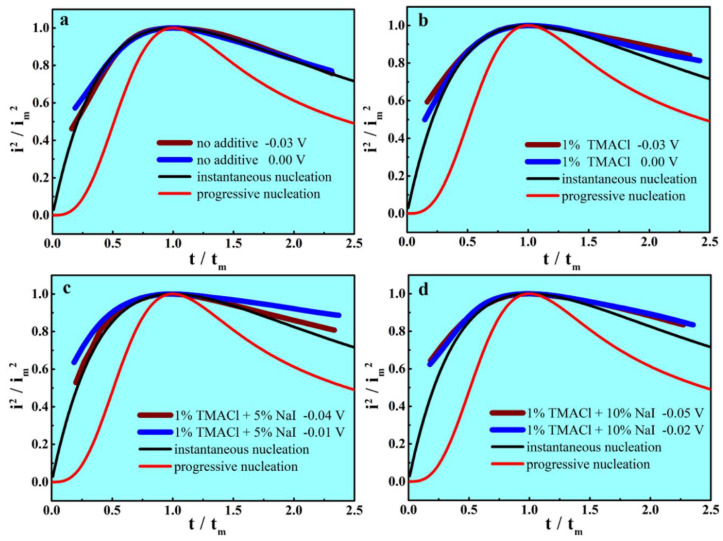
The comparison of the experimental and theoretical plots of (i/i_m_)^2^ vs (t/t_m_) in the AlCl_3_-NaCl-KCl molten salts electrolyte, (**a**) no additives, (**b**) 1% TMACl, (**c**) 1% TMACl + 5% NaI and (**d**) 1% TMACl + 10% NaI.

During the electrodeposition of metals, the three-dimensional nucleation and hemispherical growth of the initial nuclei usually occur at the same time. A total of two different models are used to analyze three-dimensional nucleation/growth qualitatively, namely ‘instantaneous’ or ‘progressive’ [37]. The instantaneous model depicts the status that all of the nucleation sites are activated simultaneously. In contrast, the progressive model depicts the status that the nucleation sites are gradually activated in the whole process of chronoamperometric experiment. The comparison of the experimental transients and the theoretical transients is adopted for the identification of nucleation models. The theoretical transients for instantaneous and progressive nucleation equations are listed as Equations (2) and (3), respectively:

(2) $${\left(\frac{i}{i_{m}}\right)}^{2}=\frac{1.9542}{t/t_{m}}{\left\{1-\exp\left[-1.2564\left(\frac{t}{t_{m}}\right)\right]\right\}}^{2}$$

(3) $${\left(\frac{i}{i_{m}}\right)}^{2}=\frac{1.2254}{t/t_{m}}{\left\{1-\exp\left[-2.3367{\left(\frac{t}{t_{m}}\right)}^{2}\right]\right\}}^{2}$$

where i represents the current density, t represents the time and i_m_ represents the maximum of the current density at t_m_ time. To determine effect of TMACl and NaI on the nucleation/growth mechanism, the data obtained from the chronoamperometric tests were normalized and compared with the theoretical transients. Figure 4 depicts the experimental and theoretical plots of (i/i_m_)^2^ vs (t/t_m_) with different composition of additives.

It is obvious that the experimental curves are mostly in accordance with the instantaneous nucleation model, which suggests that the process of Al electrodeposition could be referred to instantaneous nucleation. The addition of two additives does not affect the mechanism of Al nucleation and growth, indicating that the process of nucleation/growth exhibits irrelevance with TMACl or NaI.

### 3.4. Morphology of Al Coatings

**Figure 5:**
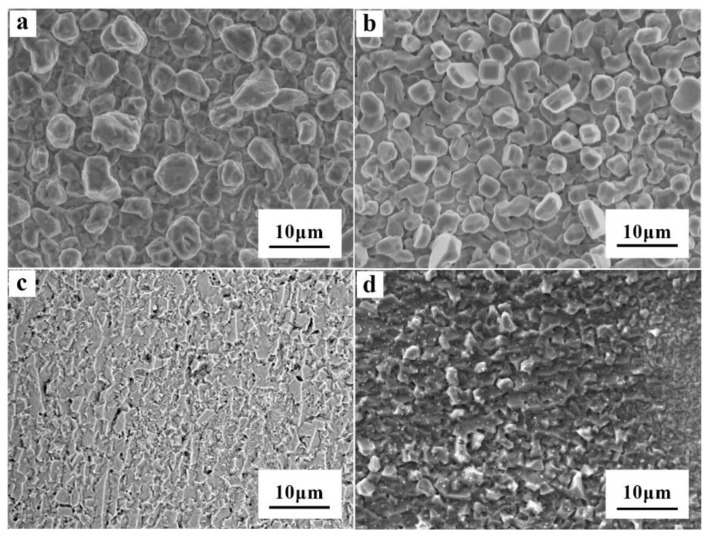
The microscopic morphologies of the aluminum electrodeposits obtained in AlCl_3_-NaCl-KCl (80-10-10 wt.%) molten salts electrolyte with different additives, (**a**) no additives, (**b**) 1% TMACl, (**c**) 1% TMACl + 5% NaI and (**d**) 1% TMACl + 10% NaI.

Al deposited samples obtained from AlCl_3_-NaCl-KCl molten salts electrolyte with varied concentration of TMACl and NaI at 150 °C were examined by SEM. The typical SEM images of the surface of Al coatings are shown in Figure 5.

As no additive was introduced, shown in Figure 5a, the surface of the Al coating was still rough, and the films were nonuniform—the average particle size was about 7 ± 3 μm. When 1 wt.% TMACl was added into the electrolyte, as shown in Figure 5b, the grains became more uniform, the average particle size became smaller, the Al coating became dense. Extra experiments proved that the addition of more TMACl at 5%, 10%, and even 15%, had a similar effect on the Al coatings, but the morphology and quality of the Al deposit did not change much. This means that more TMACl could not magnify the effect which serves as an effective additive in molten salts electrolyte or improve the quality of Al coatings effectively. However, when TMACl and NaI were both introduced, whether 1% TMACl and 5% NaI or 1% TMACl and 10% NaI (Figure 5c,d), the Al particles became smaller and homogeneous, the surface of Al coating became flat and consecutive, and the size of Al particles appeared uniform. The Al particles grew neatly, and a compact and uniform electrodeposition Al coating was obtained, where the average particle size was approximately 2 ± 1 μm. The dendritic crystal and spongy are prevented effectively, indicating that NaI is helpful to refine the particle size. On account of the experiment results of cyclic voltammetry in Section 3.2, it is reasonable to speculate that the the addition of TMACl and NaI, especially the addition of NaI, could cause electrochemical polarization. A similar trend was also found in the electrodeposition of Al with ionic liquid electrolytes containing similar ammonium halides as additives [38]. While, there were a few tiny pores on the surface of the Al coating. The gap between Al particles became narrower after TMACl and NaI were added, and finally appeared in the form of voids on the coating surface.

**Figure 6:**
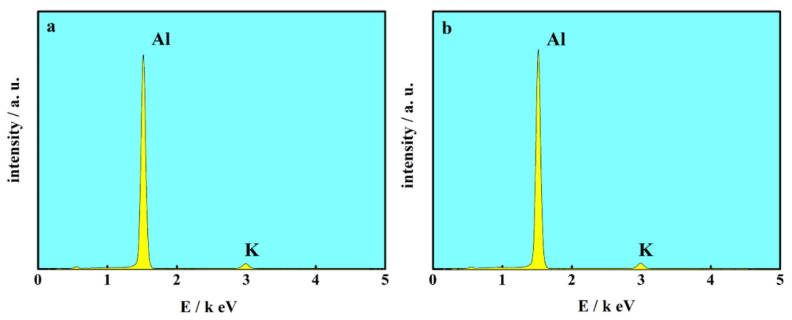
The EDXS spectra of the Al deposits obtained on a copper foil in AlCl_3_-NaCl-KCl (80-10-10 wt.%) molten salts electrolyte, (**a**) 1% TMACl, (**b**) 1% TMACl + 10% NaI.

In order to determine the elementary composition of the Al deposits, the EDXS tests were performed. The typical EDXS spectrums of the Al electrodeposits from AlCl_3_-NaCl-KCl molten salts electrolyte with additives are presented in Figure 6. The strong peak of aluminum can be clearly observed, indicating the main composition of the coating is aluminum. The tiny peak of K was also detected since there could probably be a little KCl residue from the electrolyte on the surface of Al coatings. No peak of oxygen or other element was detected after the addition of TMACl, or both TMACl and NaI (Figure 6a,b). The EDXS results proved that the electrodeposited Al coatings consisted of pure Al and were clear of the TMACl or NaI, confirming that the electrodeposition Al coatings obtained with additives were of high quality. A layer of high-purity Al coating can be obtained from AlCl_3_-NaCl-KCl molten salts electrolyte with TMACl and NaI added as additive.

**Figure 7:**
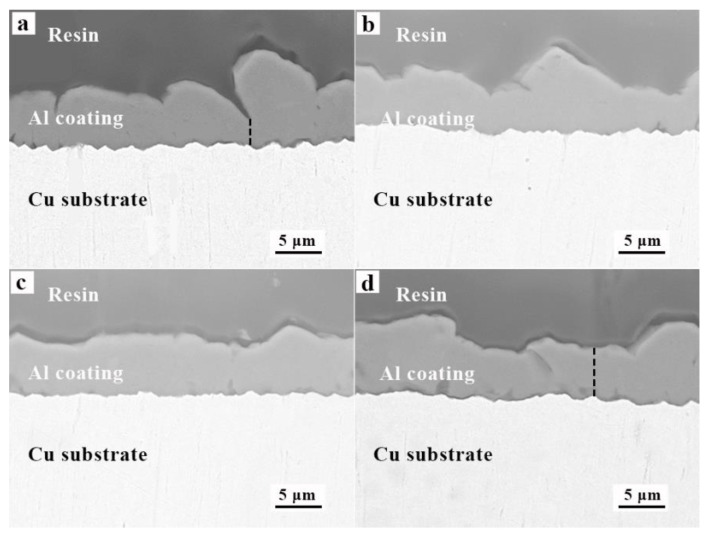
The cross-sectional morphology of the Al coating deposited from the AlCl_3_-NaCl-KCl molten salts electrolyte with different additives, (**a**) no additives, (**b**) 1% TMACl, (**c**) 1% TMACl + 5% NaI and (**d**) 1% TMACl + 10% NaI.

The cross-sectional morphology of the Al coating obtained from AlCl_3_-NaCl-KCl molten salts with no additives, 1% TMACl, 1% TMACl + 5% NaI and 1% TMACl + 10% NaI are presented in Figure 7. It shows in Figure 7a that the thickness of the electrodeposition Al coating obtained from the electrolyte without additive varies from 2.5 to 10 μm, and the average thickness of Al electrodeposits obtained was 6 ± 4 μm. The nonuniformity of the thickness means the Al coatings are not dense and compact. In contrast, after 1% TMACl + 10% NaI added into the electrolyte, the average thickness of the achieved Al coating is approximately 7 ± 2 μm. There is no gap between Al coatings and the Cu substrate indicating the electrodeposited Al coating and the Cu substrate adhere tightly. The Faraday’s law was used to calculate the cathodic current efficiency based on the mass of the electrodeposited Al, as 1% TMACl and 10% NaI were added into the electrolyte, Al coatings with a current efficiency of 99% was obtained.

**Figure 8:**
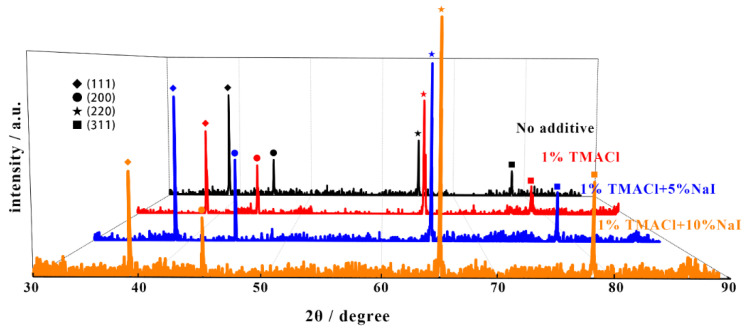
The XRD patterns of the electrodeposition Al coating obtained on a copper foil in AlCl_3_-NaCl-KCl (80–10–10 wt.%) molten salts electrolyte, without additive, 1% TMACl, 1% TMACl + 5% NaI and 1% TMACl + 10% NaI, at 150 °C.

To further characterize the microstructure of the Al coating, XRD analysis was performed and Figure 8 shows the XRD patterns of the electrodeposited Al coatings. All the strong diffraction peaks in the XRD patterns can be referred to the Al coatings and the Cu substrate. In total, four obvious diffraction peaks, (111), (200), (220), and (311), can be found from the obtained aluminum deposits. It means that the Al coatings are constituted of fcc phase of Al metal. In order to study the effect of the two additives on the orientation and grain size of Al coatings, the texture coefficient (TC) and the grain size were adopted and calculated via XRD reflections [39], and the results are listed in Table 1. As the additive was added, the grain size of the Al decreased significantly, which is in accordance with the variation tendency of particle size. The addition of TAMCl and NaI could contribute to the formation of a preferred crystallographic orientation along (220) plane and refine the grain size effectively.

### 3.5. Corrosion Behaviors

**Figure 9:**
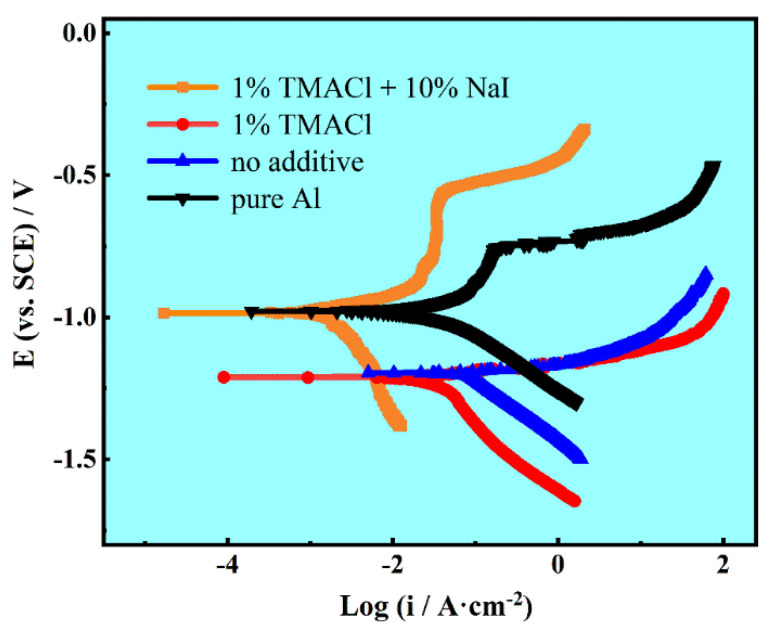
The potentiodynamic polarization curves of pure Al, Al coating electrodeposited without additive, with 1% TMACl and with 1% TMACl + 10% NaI.

The potentiodynamic polarization was performed so as to test the corrosion resistance of Al coatings obtained from AlCl_3_-NaCl-KCl molten salts electrolyte with varied concentration of additives. A cast pure Al plate was also tested as a parallel control experiment. Figure 9 presents a series of typical potentiodynamic polarization curves of the electrodeposited Al coatings with different additives, and other detail parameters calculated by the Tafel extrapolation are shown in the Table 2.

It can be seen that the Al coatings electrodeposited from AlCl_3_-NaCl-KCl molten salts electrolyte without additive present the poor corrosion resistance with the highest i_corr_ of 40.161 μA. After 1% TMACl was added in to the electrolyte, the potentiodynamic polarization parameters did not change much. It is reasonable, since the Al coatings were not dense and compact enough, the loose Al particles made the coatings easily corroded. Further, this also gives an explanation as to why there is no passive region found in these two curves. In contrast, as the 1% TMACl + 10% NaI was added, the corrosion current i_corr_ of the electrodeposited Al coatings declined dramatically to 3.625 μA, which is quite close to that of pure Al. A conspicuous passive region was observed in the potentiodynamic polarization curve, indicating that the Al coating exhibits a great corrosion resistance. It also proved that the addition of TMACl could not affect the quality of Al coatings, while the addition of TMACl and NaI could improve the Al coatings prominently.

**Figure 10:**
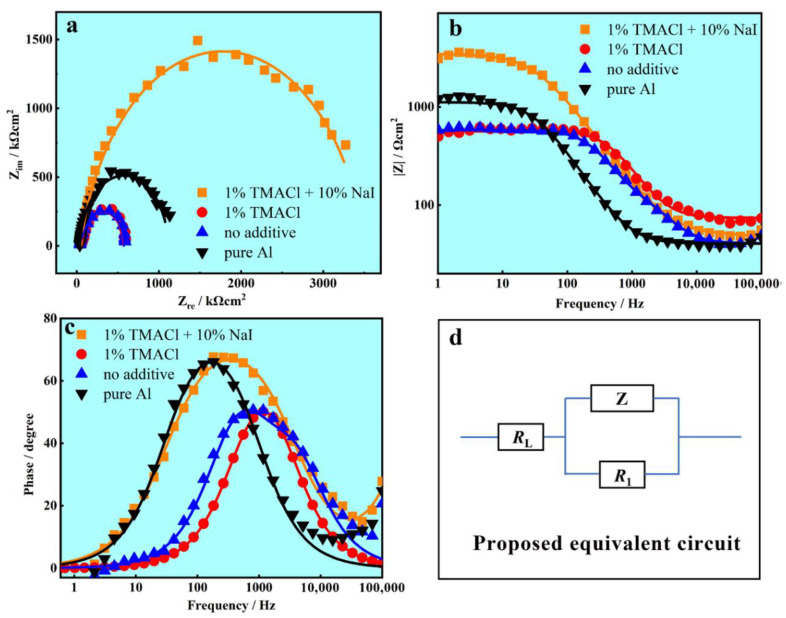
The EIS plots of pure Al, Al coating electrodeposited without additive, with 1% TMACl and with 1% TMACl + 10% NaI. (**a**) Nyquist plots, (**b**) Bode plots, (**c**) Bode-phase plots and (**d**) equivalent circuit.

The EIS tests were performed at the OCP to further evaluate the corrosion resistance of the electrodeposition Al coatings obtained from AlCl_3_-NaCl-KCl molten salts electrolyte with additive and the results of are presented in Figure 10. It is shown that the shapes of Nyquist plots of pure Al, Al coating electrodeposited without additive, with 1% TMACl and with 1% TMACl + 10% NaI were very similar, revealing that the corrosion process is mainly controlled by the charge-transfer [40]. In the Nyquist plots, the larger semicircular arc diameter means the greater resistance. Bode and Bode-phase results have proved that the Al coating achieved with 1% TMACl + 10% NaI presented the highest /Z/. Hence, the Al coating electrodeposited from AlCl_3_-NaCl-KCl molten salts electrolyte with 1% TMACl + 10% NaI exhibit a great corrosion resistance.

The equivalent circuit in accordance with the Nyquist curves is shown in Figure 10d. R_L_ is the resistance of the AlCl_3_-NaCl-KCl molten salts electrolyte at a high frequency limit. R_1_ is the polarization resistance related to the charge transfer, and Z determines the double layer capacitance. The simulation values obtained by Z-view software for the equivalent elements are shown in Table 3. Generally, a higher R_1_ reveals a lower corrosion resistance. According to Table 3, it is obvious that the Al coating electrodeposited from AlCl_3_-NaCl-KCl molten salts electrolyte with 1% TMACl + 10% NaI presented the best corrosion resistance.

## 4. Conclusions

In order to effectively reduce the volatility of the AlCl_3_-NaCl-KCl (80–10–10 wt.%) molten salts electrolyte, increase electrodeposition efficiency and improve the quality of electrodeposition Al coatings, two different additives, TMACl and NaI were introduced and investigated. The addition of TMACl could inhibit the volatilization of AlCl_3_, extending the service life of electrolyte. High quality Al coatings were obtained by electrodeposition from AlCl_3_-NaCl-KCl molten salts electrolytes at 150 °C with varied concentration of NaI and TMACl. A uniform, compact and smooth layer of electrodeposited Al films can be obtained by adding 1% TMACl + 5% NaI and 1% TMACl + 10% NaI. The electrodeposited Al coatings achieved great corrosion resistance, close to that of pure Al plate, with a corrosion current of 3.625 μA. The average particle size of the Al deposits was approximately 2 ± 1 μm. The average thickness of the electrodeposition Al coatings was approximately 7 ± 2 μm. The electrodeposition of Al on a Cu electrode in AlCl_3_-NaCl-KCl (80–10–10 wt.%) molten salts electrolyte proceeded via a three-dimensional instantaneous nucleation—the process of nucleation and growth exhibits irrelevance with NaI or TMACl. The addition of TMACl could not affect and improve the electrodeposition effectively. However, the addition of TMACl and NaI could intensify the cathodic polarization and inhibit the electrodeposition of Al. The combined effort of the two additives could increase the conductivity and facilitate to refine the particle size, contributing to the formation of a continuous, homogeneous and uniform Al coatings. They can be used as effective additives in molten salts electrolytes.

## Figures and Tables

**Table 1:** The *TC* values and grain sizes calculated from XRD reflections, Al coating electrodeposited without additive, with 1% TMACl, and with 1% TMACl + 10% NaI.

Additive	*TC*_(111)_	*TC*_(200)_	*TC*_(220)_	*TC*_(311)_	Grain Size (nm)
No additives	17.89%	15.11%	45.73%	21.27%	417
1% TMACl	9.76%	13.49%	60.73%	16.02%	401
1% TMACl + 5% NaI	10.51%	14.04%	57.49%	17.46%	395
1% TMACl + 10% NaI	5.72%	7.77%	62.24%	24.27%	363

**Table 2:** Electrochemical parameters calculated from the potentiodynamic polarization curves in 3.5 wt.% NaCl solution.

Specimens	E_corr_ (vs. SCE)/V	i_corr_/μA·cm^−2^
Al coatings electrodeposited with 1% TMACl + 10% NaI.	−0.985	3.625
Al coatings electrodeposited with 1% TMACl	−1.211	38.768
Al coatings electrodeposited with No additive	−1.196	40.161
Pure Al	−0.978	35.140

**Table 3:** EIS data for pure Al, Al coating electrodeposited without additive, with 1% TMACl and with 1% TMACl + 10% NaI, in 3.5% NaCl solutions.

Specimens	*R*_L_ (Ω cm^2^)	*Z* (μF cm^2^)	*R*_1_ (Ω cm^2^)
Al coatings electrodeposited with 1% TMACl + 10% NaI.	55.48	10.97	3258
Al coatings electrodeposited with 1% TMACl	74.03	76.42	488.5
Al coatings electrodeposited with No additive	44.47	10.66	52.32
Pure Al	41.13	46.12	1059
